# Aberrant brain gray matter and functional networks topology in end stage renal disease patients undergoing maintenance hemodialysis with cognitive impairment

**DOI:** 10.3389/fnins.2022.967760

**Published:** 2022-08-10

**Authors:** Jiahui Zheng, Xiangxiang Wu, Jiankun Dai, Changjie Pan, Haifeng Shi, Tongqiang Liu, Zhuqing Jiao

**Affiliations:** ^1^Department of Radiology, The Affiliated Changzhou No. 2 People’s Hospital of Nanjing Medical University, Changzhou, China; ^2^GE Healthcare, MR Research China, Beijing, China; ^3^Department of Nephrology, The Affiliated Changzhou No. 2 People’s Hospital of Nanjing Medical University, Changzhou, China; ^4^School of Computer Science and Artificial Intelligence, Changzhou University, Changzhou, China

**Keywords:** end-stage renal disease, resting-state functional magnetic resonance imaging, diffusion kurtosis imaging, graph theoretical analysis, predict

## Abstract

**Purpose:**

To characterize the topological properties of gray matter (GM) and functional networks in end-stage renal disease (ESRD) patients undergoing maintenance hemodialysis to provide insights into the underlying mechanisms of cognitive impairment.

**Materials and methods:**

In total, 45 patients and 37 healthy controls were prospectively enrolled in this study. All subjects completed resting-state functional magnetic resonance imaging (rs-fMRI) and diffusion kurtosis imaging (DKI) examinations and a Montreal cognitive assessment scale (MoCA) test. Differences in the properties of GM and functional networks were analyzed, and the relationship between brain properties and MoCA scores was assessed. Cognitive function was predicted based on functional networks by applying the least squares support vector regression machine (LSSVRM) and the whale optimization algorithm (WOA).

**Results:**

We observed disrupted topological organizations of both functional and GM networks in ESRD patients, as indicated by significantly decreased global measures. Specifically, ESRD patients had impaired nodal efficiency and degree centrality, predominantly within the default mode network, limbic system, frontal lobe, temporal lobe, and occipital lobe. Interestingly, the involved regions were distributed laterally. Furthermore, the MoCA scores significantly correlated with decreased standardized clustering coefficient (γ), standardized characteristic path length (λ), and nodal efficiency of the right insula and the right superior temporal gyrus. Finally, optimized LSSVRM could predict the cognitive scores of ESRD patients with great accuracy.

**Conclusion:**

Disruption of brain networks may account for the progression of cognitive dysfunction in ESRD patients. Implementation of prediction models based on neuroimaging metrics may provide more objective information to promote early diagnosis and intervention.

## Introduction

End-stage renal disease (ESRD) is the final stage of chronic kidney disease (CKD). Treatment options for ESRD include continuous hemodialysis, peritoneal dialysis, and kidney transplantation. ESRD patients are at high risk of developing cognitive impairment (CI), especially in patients who have received continuous hemodialysis, with a prevalence of 30–60% (Bugnicourt et al., 2013; Kalantar-Zadeh et al., 2021). Impaired domains include overall cognition, executive function, memory, motor, and attention (van Zwieten et al., 2018). CI in ESRD patients is associated with negative outcomes, including non-adherence to drug treatment and increased rate of suicide (Agganis et al., 2010; Natale et al., 2019). However, the underlying neuropathology of CI in ESRD patients remains largely unknown. Therefore, investigating the neuropathological alterations leading to CI would help to understand the potential mechanisms contributing to CI, which would be beneficial in preparing treatment plans for ESRD patients.

Magnetic resonance imaging (MRI) is a non-invasive technique that can provide structural and functional information of the brain. It has been widely used to study the neurological changes in CI-related diseases, including Parkinson’s disease (Donzuso et al., 2021), silent cerebral infarction (Luo et al., 2015), Alzheimer’s disease (AD) (Chandra et al., 2019), and multiple sclerosis (Zhang J. et al., 2021). Voxel-based morphometry (VBM) is a valuable tool for assessing brain volume changes on a voxel-by-voxel basis (Pezzoli et al., 2021). Recently, Jin et al. (2020) reported that a predominant decrease in gray matter (GM) volume is associated with CI in ESRD patients by using VBM based on structural MRI technologies. Diffusion tensor imaging (DTI) is a valuable MRI technique that can estimate the microstructure of tissues by probing the diffusion process of water molecules (Basser et al., 1994). Through the use of DTI, previous studies found that disruption of white matter (WM) integrity correlated with impaired kidney function and CI in ESRD patients (Chou et al., 2019; Park et al., 2020; Mu et al., 2021). Functional imaging techniques, such as resting-state functional magnetic resonance imaging (rs-fMRI) and arterial spin labeling (Zheng et al., 2016), have been utilized to detect brain functional alterations in ESRD. Recently, Li et al. (2021) investigated disrupted neurovascular coupling in ESRD patients undergoing hemodialysis and revealed it to be a potential neural mechanism for CI. Moreover, rs-fMRI can detect low-frequency (0.01–0.08 Hz) fluctuations in blood-oxygen-level-dependent (BOLD) signals and can be used to investigate spontaneous neural activity at rest (Barkhof et al., 2014). In hemodialysis patients, impaired functional integrity of extensive brain regions has been measured by using multiple analytical methods based on rs-fMRI, including regional homogeneity (ReHo), amplitude of low frequency fluctuation (ALFF), and functional connectivity (FC) (Li et al., 2018; Chen et al., 2020; Hu et al., 2020; Guo et al., 2021). Although the above results were inconsistent due to the diverse population cohorts and methodologies used, a correlation between functional abnormalities and cognitive dysfunction was indicated.

Complex structural and functional brain networks can provide a physiological basis for information processing among neural elements and mental representation (McIntosh, 2000; Friston, 2002). Diffusion MRI and fMRI are the most extensively used non-invasive imaging methods for reconstructing structural and functional networks, with network nodes representing brain regions and network edges representing structural or functional connectivity (Suárez et al., 2020). Currently, graph theory can be used to evaluate the architecture, development, and evolution of brain networks systematically and quantitatively by quantitatively analyzing topological properties (Reijneveld et al., 2007; Bullmore and Sporns, 2009). Recent studies have used graph theory to detect the complexity of brain networks in various neurological diseases, including schizophrenia, AD, depressive disorder, acute brainstem ischemic stroke, and epilepsy (Jiang et al., 2017; Faskhodi et al., 2018; Shi et al., 2021; Zhang L. et al., 2021; Zhang Y. et al., 2021). In these disorders, the emergence of clinical symptoms or functional impairment was related to the disrupted integration of spatially distributed brain regions in structural and functional networks (Bullmore and Sporns, 2009). Similarly, the use of graph theory with rs-fMRI and DTI has identified brain aberrations in functional and WM networks correlating with cognitive function in ESRD patients (Ma C. et al., 2020; Wu et al., 2020). However, no studies to date have investigated the relationship between topological characteristics of GM networks and cognitive function in ESRD patients.

Diffusion kurtosis imaging (DKI), an extension of the DTI model, was developed to sensitively quantify non-Gaussian water diffusions in a voxel (Jensen et al., 2005). Thus, DKI provided a useful way to investigate abnormalities in both GM and WM, where DTI was less capable. Currently, DKI has been used as a practical clinical application in neuroscience research, including normal brain tissues, brain trauma, brain tumors, AD, schizophrenia, and Parkinson’s disease (McKenna et al., 2019; Bingbing et al., 2020; Goghari et al., 2021; Hempel et al., 2021; Stenberg et al., 2021). These studies supported the possibility that DKI may be a sensitive imaging biomarker in many neurological diseases. However, these studies only investigated kurtosis values in regional brain areas, ignoring the coordination of heterogeneous DKI properties between different brain regions. A recent study focused on changes in both GM and WM networks based on DKI in AD found that DKI network damage was related to cognitive performance (Cheng et al., 2018). However, few studies have applied DKI to investigate CI in ESRD patients. More specifically, no studies have applied DKI to assess GM networks in ESRD patients.

In this study, we used DKI and rs-fMRI to construct GM functional networks to investigate potential aberrant mechanisms leading to CI in ESRD patients. Further, we tentatively predicted the cognitive function of ESRD patients using topological properties as features based on the individual level. The least squares support vector regression machine (LSSVRM) was used to build a prediction model, and the whale optimization algorithm (WOA) was used to optimize model parameters (Zhang et al., 2022). In conclusion, our study attempted to improve the possibility of early diagnosis and neuroprotective treatments for ESRD patients by using predictive models based on neuroimaging techniques.

## Materials and methods

### Participants

This study was approved by the Ethics Committee of The Affiliated Changzhou No. 2 People’s Hospital of Nanjing Medical University (Number: KY039-01). All participants provided informed consent to participate in the study. All subjects were right-handed and fully capable of completing the Montreal cognitive assessment scale (MoCA) test independently.

Between February 2020 and December 2021, 45 ESRD patients (22 male and 23 female, mean age 49.56 ± 8.02 years) from our hospital were prospectively recruited into our patient group. Inclusion criteria for the patient group included: (1) clinically diagnosed ESRD (estimated glomerular filtration rate less than 15 mL/min/1.73 m^2^); (2) receiving regular maintenance hemodialysis lasting longer than 3 months (three times a week at the hemodialysis center); (3) age between 30 and 65 years; and (4) MoCA score less than 26. Exclusion criteria included: (1) history of other neuropsychiatric disorders; (2) history of head trauma, intracranial tumors, or cerebral infarction; (3) renal transplant history; (4) drug or alcohol abuse history; (5) contraindications to MRI, such as claustrophobia, dental fixtures, and other exogenous objects in the head; and (6) obvious head motion artifact.

In addition, 37 healthy controls (HCs) (19 male and 18 female, mean age 47.30 ± 7.20 years) without renal disease and other known disorders and with MoCA scores higher than 26 were enrolled at the same time as the control group. Both groups were matched based on age, gender, and education years. The exclusion criteria for HCs were the same as that for the patient group.

### Neuropsychological assessment

MoCA testing was administered to measure the overall cognitive status (out of a possible 30 points), with patients scoring less than 26 diagnosed with CI (Nasreddine et al., 2005). MoCA testing was administered by a well-trained clinical neuropsychologist within 2 h prior to MR scanning.

### Laboratory tests

Laboratory tests for all ESRD patients were conducted within 24 h prior to MR scanning and included white and red cell counts, hemoglobin, hematocrit, fasting glucose, urea nitrogen, creatinine, uric acid, cholesterol, triglyceride, and calcium measurements. Laboratory tests were not performed for patients in the HC group.

### Research framework

**FIGURE 1:**
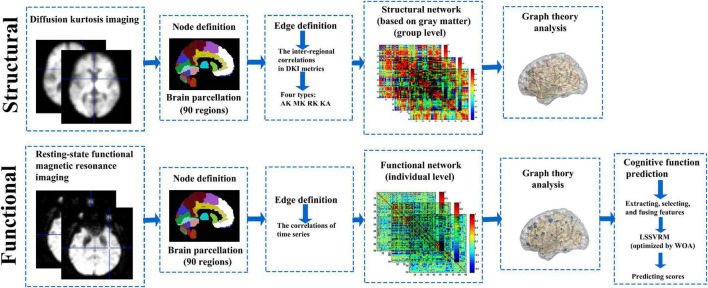
Research framework. GM and functional brain networks were explored using graph theory through the following four steps: (1) define the network nodes. These were defined as anatomically defined regions of the AAL template; (2) estimate a continuous measure of association between nodes. These were the interregional correlations in DKI metrics and time series data; (3) generate an association matrix by compiling all pairwise associations between nodes, and apply a threshold to each element of this matrix to produce a binary adjacency matrix; and (4) calculate the network parameters of interest in this graphical model of the brain networks and compare them to the equivalent parameters of a population of random networks. GM, gray matter; AAL template, automated anatomical labeling template; DKI, diffusion kurtosis imaging.

Research framework is shown in Figure 1.

### Magnetic resonance imaging data acquisition

Magnetic resonance imaging data were acquired using a 3.0T magnetic resonance scanner (Discovery MR750W, General Electric Medical Systems, United States, Milwaukee, WI), equipped with a standard 32-channel head and spine combined coil. All participants were asked to stay still and awake and to keep their eyes closed during the entirety of scanning. Earplugs were used to alleviate the noise from the MR scanner. Foam pads were added on both sides of the head to reduce head motion. High-resolution anatomic T1-weighted images were acquired with the three-dimensional brain volume imaging (3D-BRAVO) sequence [parameters: 152 slices; slice thickness = 1.2 mm (no gap); repetition (TR) = 8.2 ms; echo time (TE) = 3.2 ms; flip angle (FA) = 12°; matrix = 256 × 256; field of view (FOV) = 240 mm × 240 mm; whole scanning time = 3 min 57 s]. rs-fMRI data were acquired with the gradient-recalled echo-planar imaging (GRE-EPI) sequence (parameters: 33 slices; 240 time-points; slice thickness = 4 mm; TR = 2,000 ms; TE = 40 ms; FA = 90°; matrix = 64 × 64; FOV = 240 mm × 240 mm; whole scanning time = 8 min 12 s). DKI data were acquired using a single-shot echo-planar imaging (SS-EPI) sequence, with 3b-values (b = 0, 1,000, 2,000 s/mm^2^) along 30 diffusion gradient directions (parameters: NEX = 2, slice thickness = 3.6 mm (no gap); TR = 6,500 ms; TE = 95.8 ms; matrix = 128 × 128; FOV = 240 mm × 240 mm; whole scanning time = 14 min 43 s).

### Image preprocessing

#### Diffusion kurtosis imaging data

Diffusion kurtosis imaging data were preprocessed using the FMRIB Software Library (FSL),^1^ Diffusion Kurtosis Estimator (DKE),^2^ and Statistical Parametric Mapping 8 (SPM8)^3^ software. First, DKI data in DICOM format were converted into 4DNIFTI format. Data were corrected for head movement, eddy, and gradient distortion with b = 0 image as a reference using FSL. Then, DKI parameters including mean kurtosis (MK), axial kurtosis (AK), radial kurtosis (RK), and kurtosis anisotropy (KA) were extracted using DKE with the quadratic programming-based (CLLS-QP) algorithm (Tabesh et al., 2011). Next, after registering each subject’s 3D-T1w anatomic images with the standard Montreal Neurologic Institute (MNI) template, b = 0 images were registered with the standardized 3D-T1w anatomic images using SPM8 with a non-linear co-registration technique. The resulting transformation matrix was used for normalizing all DKI parameter maps. Finally, normalization and smoothing (FWHM = 6 mm) for DKI parameters were conducted using SPM8.

#### Resting-state functional magnetic resonance imaging data

Resting-state functional magnetic resonance imaging data preprocessing was performed with the data processing assistant for resting-state fMRI (DPARSF-V2.3)^4^ based on MATLAB 2013. First, after converting data from the DICOM format to the NIFTI format, the first 10 timepoints were discarded to allow for steady-state longitudinal magnetization. Slice timing and head motion correction were conducted for the remaining 230 timepoints collected (subjects were excluded if their head movement was more than 3 mm or 3°). Next, spatial normalization was performed based on unified segmentation to the structural T1 image, and the functional image was warped into the standard space of the (MNI) template (resampling voxel size = 3 mm × 3 mm × 3 mm). Then, spatial smoothing in reference to an isotropic Gaussian kernel (full width at half maximum [FWHW] = 6 mm), detrending, and temporal filtering (bandpass, 0.01–0.08 Hz) were performed. Finally, nuisance covariates including cerebrospinal fluid signals, WM signals, and Friston-24 head motion parameters were regressed out of the data.

### Network construction

#### Gray matter network construction

Gray matter networks were constructed at the group level based on each DKI parameter measurement. The structural connections of the GM network were defined as statistical correlations between pairs of kurtosis values from brain nodes. Nodes were defined as the parcellation of a whole-brain conducted according to the Automated Anatomical Labeling (AAL) atlas, which divided the brain into 90 regions including 78 cortical regions and 12 subcortical regions. These regions were regarded as the nodes of the GM network. For edge definition, regional kurtosis values for every region were extracted to calculate the Pearson’s correlation coefficients across individuals for each pair of brain regions to obtain the correlation matrix (90 × 90) of each group (Cheng et al., 2018). Then, a parameter matrix for each DKI metric was assigned to the HC and ESRD patient groups (MK, AK, RK, and KA).

#### Functional network construction

Functional networks for each participant were constructed using the GRETNA toolbox.^5^ Nodes were defined (similar to the GM network) according to the AAL atlas (90 regions). Edge definitions were calculated using the mean time series for every region to calculate the Pearson’s correlation coefficients for each pair of regions, which were used to obtain the correlation matrix (90 × 90). Then, Fisher’s Z transformation was performed to increase the normality of the matrix.

### Network analysis

#### Network threshold selection

Using the matrix sparsity as the threshold, the matrix was binarized. The whole range of sparsity thresholds of the GM network was set to 0.06–0.40 with an interval of 0.01 (the sparsity threshold was selected to ensure that all resultant networks have the same number of nodes and edges) (Cheng et al., 2018). The whole range of sparsity thresholds of the functional network was set to 0.1–0.4, with an interval of 0.01 (the values of small-worldness of all participants were checked more than once in order to avoid the selection of a threshold range too wide to produce connected nodes and networks with small-worldness features) (Wu et al., 2020).

#### Network metrics

For each sparsity threshold, global and nodal measures were calculated. Global measures included global efficiency (Eg), local efficiency (Eloc), mean clustering coefficient (Cp), characteristic path length (Lp), standardized clustering coefficient (γ), standardized characteristic path length (λ), and small-world properties (σ). Nodal measures included nodal efficiency (Ne) and degree centrality (Dc). Areas under the curve (AUCs) of the topological parameters of the functional network were calculated within the whole sparsity threshold.

## Statistical analysis

### Group differences between demographic data, clinical characteristics, and MoCA scores

Demographics, clinical data, and MoCA scores were analyzed using specific software (SPSS version 25.0; SPSS, Chicago, Illinois). Chi-squared test, two-sample independent Student’s *t*-test, and Mann-Whitney U test were used to compare gender-based differences and quantitative data between the groups. A statistical significance level was set at *P* < 0.05.

### Group differences between all imaging parameters

A two-sample *t*-test was performed based on GRETNA to detect differences in network measures between the two groups with age, gender, and education years used as covariates. We set the statistical threshold with false discovery rate criterion (FDR)- corrected *P* < 0.05.

### Correlation analysis

Pearson’s correlation analysis was performed based on GRETNA to detect the relationships between significant topological parameters of the functional network and MoCA scores in ESRD patients with age, gender, and education years used as covariates (*P* < 0.05 and corrected by FDR).

## Prediction model construction

Least squares support vector regression machine is an improvement of support vector regression machine. To improve prediction efficiency and accuracy, LSSVRM changed the inequality constraint and the solution of quadratic programming problem into equality constraint and the solution of linear equations, respectively (Liu et al., 2019). Then, to improve operating efficiency, we optimized selection strategy of kernel function parameters by introducing WOA into LSSVRM (Zhang et al., 2018).

Optimized LSSVRM was applied to predict cognitive function based on functional networks. Functional network measures (global and nodal) significantly correlated (*P* < 0.05 and corrected by FDR) with MoCA scores were selected as features. The selected features were fused to build LSSVRM, using two methods: [a] only global measures were fused; [b] both global and nodal measures were fused. Then, selected measures with corresponding MoCA scores of 45 ESRD patients were used as a data set by the leave-one-out method. For our data, leave-one-out method was optimized empirically. The mean square error (MSE), root mean square error (RMSE), mean absolute error (MAE), and mean absolute percentage error (MAPE) were chosen as testing standards to assess the accuracy of models. The lower the MSE, RMSE, MAE, or MAPE, the better the model’s prediction accuracy.

## Results

### Demographic, clinical, and neuropsychological results

No significant differences were observed in the age, gender, and education years between the participants in the two groups (*P* > 0.05). When compared to the HC group, patients in the ESRD group had significantly lower MoCA scores (*P <* 0.001) (Table 1).

**TABLE 1:** Demographic and clinical characteristics and neuropsychological test scores.

Variable	ESRD (*n* = 45)	HC (*n* = 37)	Statistic value	*P*-value
Demographic				
Age, years	49.56 ± 8.02	47.84 ± 6.71	*T* = 1.038	*P* = 0.303
Gender, male (%)	22 (49)	19 (51)	*x*^2^ 0.032	*P* = 0.858
Education, years (25%, 75%)	12 (9, 15)	12 (8, 15)	*Z* = -0.932	*P* = 0.351
Neuropsychological scores				
MoCA scores (25%, 75%)	21 (20, 23)	27 (26, 28)	*Z* = -7.807	*P* < 0.001*
Laboratory data				
Systolic pressure, mmHg	150.38 ± 27.00	-	-	-
Diastolic pressure, mmHg	91.44 ± 16.17	-	-	-
White cell count, 10^9/L	5.90 ± 2.17	-	-	-
Red cell count, 10^12/L	4.37 ± 4.21	-	-	-
Hemoglobin, g/L	107.60 ± 18.20	-	-	-
Hematocrit, %	37.17 ± 26.60	-	-	-
Fasting glucose, mmol/L	5.42 ± 1.89	-	-	-
Urea nitrogen, mmol/L	19.83 ± 8.39	-	-	-
Creatinine, μmol/L	867.49 ± 411.83	-	-	-
Uric acid, μmol/L	343.73 ± 134.11	-	-	-
Cholesterol, mmol/L	4.17 ± 1.57	-	-	-
Triglyceride, mmol/L	1.66 ± 0.89	-	-	-
Calcium, mmol/L	2.25 ± 0.21	-	-	-

*Significantly difference.

*x*^2^, Chi-square test; *T*, two-sample *t*-test; *Z*, Mann-Whitney U test;MoCA, Montreal cognitive assessment scale.

### Group differences of network measures

#### Gray matter network

**FIGURE 2:**
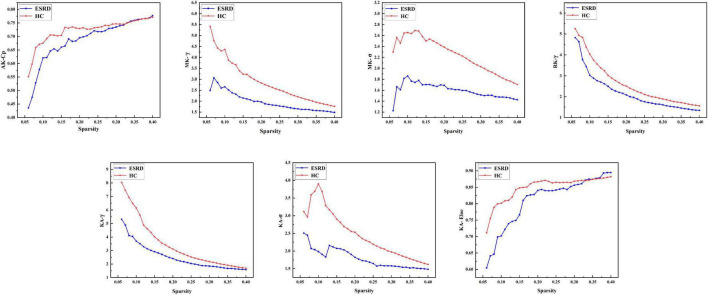
Gray matter networks. Topological measures of gray matter networks with significant between-group differences were showed. Healthy controls are shown in red, and ESRD patients are shown in blue. AK, axial kurtosis; MK, mean kurtosis; RK, radial kurtosis; KA, kurtosis anisotropy; Cp, mean clustering coefficient; γ, standardized clustering coefficient; λ, standardized characteristic path length; σ, small-world properties; Eloc, local efficiency.

The images of the group-level interregional correlation matrices using DKI metrics of AK, MK, RK, and KA are shown in Figure 2, and the between-group differences of global topological properties are presented in Table 2 and shown in Figure 2. The small-world organization of GM networks with AK, MK, RK, and KA metrics in the ESRD and HC groups was identified over a wide range of sparsity (0.06–0.4). The small-worldness values (σ = γ/λ) were larger than 1. However, when compared with the HC group, decreased topological properties of the GM network were found in ESRD patients. Decreased Cp was found in the GM network with the AK metric; decreased γ and σ were found in the GM network with the MK metric; decreased γ was found in the GM network with the RK metric; and decreased γ, σ, and Eloc were found in the GM network with the KA metric (*P* < 0.05; corrected by FDR).

**TABLE 2:** Significant between-group differences of topological parameters of gray matter (GM) network.

Structural network	Parameters	ESRD	HC	*t* value	*P*-value
AK	Cp	0.687 ± 0.081	0.722 ± 0.046	-2.194	0.028
MK	γ	1.960 ± 0.418	2.866 ± 0.945	-5.190	<0.001
	σ	1.596 ± 0.131	2.240 ± 0.310	-6.311	<0.001
RK	γ	2.183 ± 0.884	2.624 ± 1.046	-3.749	<0.001
KA	γ	2.519 ± 0.963	3.423 ± 1.788	-2.632	0.010
	σ	1.790 ± 0.277	2.459 ± 0.660	-5.532	<0.001
	Eloc	0.811 ± 0.078	0.848 ± 0.039	-2.569	0.011

Data are expressed as mean ± SD. Corrected by false discovery rate criterion (FDR) and set at *p* < 0.05.

AK, axial kurtosis; MK, mean kurtosis; RK, radial kurtosis; KA, kurtosis anisotropy; Cp, mean clustering coefficient; γ, standardized clustering coefficient; λ, standardized characteristic path length; σ, small-world properties; Eloc, local efficiency.

#### Functional network

**FIGURE 3:**
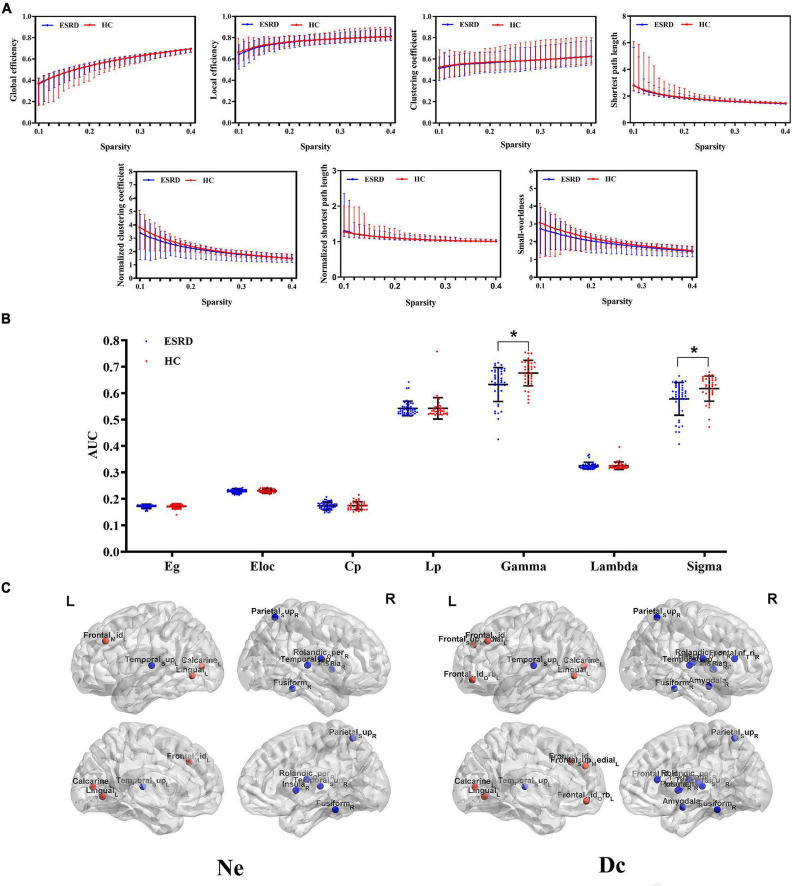
Functional networks. **(A)** Global topological measures of the whole-brain functional network were demonstrated within a whole range of sparsity values (0.1–0.4). Both groups exhibited small-world properties, with γ > 1, λ ≈ 1, and σ > 1; **(B)** Between-group differences of global topological measures of the functional network. The bottom dot-plots represent individual data points, averages (transverse line), and standard deviation (vertical lines). Healthy controls are shown in red, and ESRD patients are shown in blue; **(C)** Three-dimensional cerebral maps of nodal efficiency and nodal degree centrality. Red dots indicate brain regions with increased nodal parameters, and blue dots indicate brain regions with decreased nodal parameters.

The between-group differences of topological properties are shown in Table 3 and Figure 3. Global measures: both the ESRD and HC groups demonstrated economical small-world properties (γ greater than 1, λ approximately 1, and σ greater than 1). However, in ESRD patients, significantly decreased γ and σ were observed in the functional network (*P* < 0.05; corrected by FDR). There were no significant differences in Eg, Eloc, Cp, and Lp between the two groups (*P* > 0.05; corrected by FDR). Nodal measures: regions with significantly changed Ne and Dc (9 and 15 regions, respectively) were identified in the ESRD group and distributed laterally (*P* < 0.05; corrected by FDR), which were mainly involved in the default mode network, limbic system, frontal lobe, temporal lobe, and occipital lobe. Specifically, all right regions showed significantly decreased Ne and Dc (right-lateralized), and most left regions showed increased Ne and Dc (left-lateralized), with the left superior temporal gyrus having decreased Ne and Dc.

**TABLE 3:** Significant between-group differences of topological parameters of functional networks.

Functional network	Parameters	AAL	ESRD	HC	*t* value	*P*-value
Global measures	γ		0.633 ± 0.064	0.676 ± 0.048	-3.205	0.002
	σ		0.579 ± 0.062	0.617 ± 0.048	-3.015	0.003
Nodal measures	Nodal efficiency					
	Frontal_Mid_L	7	0.180 ± 0.013	0.173 ± 0.012	2.272	0.009
	Calcarine_L	43	0.168 ± 0.015	0.162 ± 0.010	2.155	0.012
	Lingual_L	47	0.169 ± 0.014	0.161 ± 0.016	2.322	0.018
	Temporal_Sup_L	81	0.175 ± 0.016	0.182 ± 0.015	-2.145	0.012
	Rolandic_Oper_R	18	0.172 ± 0.015	0.179 ± 0.012	-2.392	0.006
	Insula_R	30	0.175 ± 0.016	0.182 ± 0.013	-2.449	0.004
	Fusiform_R	56	0.158 ± 0.014	0.168 ± 0.013	-3.283	<0.001
	Parietal_Sup_R	60	0.163 ± 0.017	0.171 ± 0.013	-2.332	0.004
	Temporal_Sup_R	82	0.171 ± 0.016	0.179 ± 0.013	-2.490	0.004
	Degree centrality					
	Frontal_Mid_L	7	7.886 ± 2.143	6.936 ± 1.929	2.069	0.014
	Frontal_Mid_Orb_L	9	7.483 ± 1.841	6.169 ± 1.690	3.036	<0.001
	Frontal_Sup_Medial_L	23	7.113 ± 1.924	6.243 ± 1.231	2.220	0.003
	Calcarine_L	43	6.579 ± 1.920	5.767 ± 1.670	2.065	0.019
	Lingual_L	47	6.627 ± 1.901	5.794 ± 1.884	1.997	0.022
	Temporal_Sup_L	81	7.127 ± 1.907	7.917 ± 1.781	-2.179	0.020
	Frontal_Inf_Tri_R	14	5.991 ± 1.752	6.737 ± 1.830	-2.037	0.021
	Rolandic_Oper_R	18	6.705 ± 1.616	7.607 ± 1.516	-2.601	0.003
	Insula_R	30	7.110 ± 1.778	8.031 ± 1.535	-2.518	0.004
	Amygdala_R	42	6.427 ± 1.551	7.266 ± 1.894	-2.237	0.011
	Fusiform_R	56	5.100 ± 1.480	6.239 ± 1.570	-3.254	<0.001
	Parietal_Sup_R	60	5.659 ± 1.941	6.572 ± 1.476	-2.246	0.005
	Putamen_R	74	6.755 ± 1.765	7.589 ± 1.425	-2.361	0.004
	Heschl_R	80	5.811 ± 1.562	6.745 ± 1.719	-2.396	0.003
	Temporal_Sup_R	82	6.728 ± 1.734	7.787 ± 1.373	-2.849	0.001

Data are expressed as mean ± SD. Corrected by false discovery rate criterion (FDR) and set at *p* < 0.05.

γ, standardized clustering coefficient; σ, small-world properties; AAL, automated anatomical labeling.

### Correlation analysis

**FIGURE 4:**
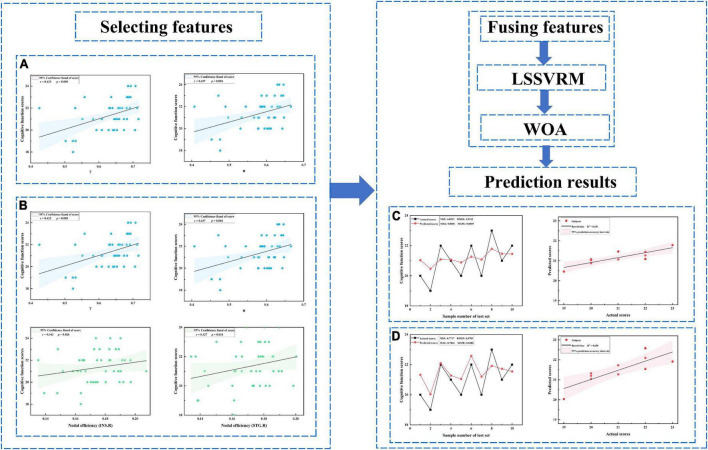
Prediction results. **(A)** Selected features including only global measures. **(B)** Selected features including both global and nodal measures. **(C)** Prediction results for panel **(A)**. **(D)** Prediction results for panel **(B)**. LSSVRM, least squares support vector regression machine; WOA, whale optimization algorithm.

In the ESRD group, decreased γ and σ of the functional network positively correlated with MoCA scores (*r* = 0.42, *r* = 0.44, respectively; *P* < 0.05, corrected by FDR). Further, a decreased Ne of the functional network in the right insula and the right superior temporal gyrus was positively correlated with MoCA scores (*r* = 0.34, r = 0.33, respectively; *P* < 0.05, corrected by FDR) (Figure 4).

### Prediction results

Optimized LSSVRM based on functional networks predicted the cognitive function of ESRD patients with great accuracy. When using selected global measures as features, MSE, RMSE, MAE, and MAPE between the actual scores and predicted scores were 0.85, 0.92, 0.84, and 4.05%, respectively, with an R-squared (R^2^) of 0.69. When both selected global and nodal measures were used as features, the MSE, RMSE, MAE, and MAPE between the actual scores and predicted scores were 0.77, 0.88, 0.78, and 3.80%, respectively, with an R^2^ value of 0.65 (Figure 4).

## Discussion

The current study investigated the changes of GM and functional network topology in ESRD patients based on DKI and rs-fMRI technologies. The changed network topology was further used to predict ESRD patient cognitive function. Our results showed the following: (1) both the GM and functional networks presented small-world characteristics in ESRD patients and HC controls; (2) both the GM and functional networks tended to be less optimized in ESRD patients, indicated by significantly decreased global properties and aberrant nodal properties; (3) functional network construction demonstrated a lateralized distribution of changed Ne and Dc in ESRD patients compared to HCs; and (4) in the functional network, decreased γ, σ, and Ne in the right insula and superior temporal gyrus were significantly correlated with cognitive dysfunction in ESRD patients. Interestingly, related topological parameters could be selected as features to predict cognitive function in ESRD patients in our study.

Graph theory analysis can quantitatively analyze the segregation and integration of brain networks (Rubinov and Sporns, 2010). Functional segregation represents the ability of functional specialization within interconnected brain areas and is measured by Cp, γ, and Eloc. Functional integration represents the ability of global communication within distributed brain regions and is measured by Lp, λ, and Eg. Consistent with previous work, we demonstrated the common small-world organization of both GM and functional networks in ESRD patients and HCs (Yue et al., 2021). However, in our study, changed topological parameters of both GM and functional networks were found in ESRD patients compared with HCs. The data revealed a downward trend of the highly optimized balance of small-world network organization. Specifically, our results indicate that this organization may have shifted toward a hemodialysis-associated network randomization, to some extent (Bullmore and Sporns, 2012).

Based on the group level, disruption of GM networks within four DKI metrics was found in the ESRD group, as indicated by decreased global measures. In a previous study, similar disruption of the GM network had been observed in AD (Cheng et al., 2018). In addition, the changed topological properties were thought to be caused by the pathology of AD, including astrogliosis, microglial activation, vascular hyalinization, and axonal loss (Cheng et al., 2018). Thus, we assumed that alterations of GM networks in ESRD patients could be linked to changes in brain microstructure. Decreased γ and σ were found in the GM network with MK, which may reflect reduced complexity of the brain microstructure (Steven et al., 2014). Decreased Cp and γ were also found in the GM network with AK and RK, which may indicate the loss of nerve axons and a breakdown of myelin membranes (Hui et al., 2008). Furthermore, the GM network with KA involved more topological parameters in our study, which may suggest that changes in fiber bundle crossover regions were more severe. To some extent, this result was consistent with a previous study focused on the WM network demonstrating the disrupted fiber integrity (Mu et al., 2021). Interestingly, GM networks with four DKI metrics showed various aspects of disrupted microstructure, indicating significantly impaired functional segregation and relatively preserved functional integration of GM networks in ESRD patients. These results suggest that DKI may be sensitive enough to detect microstructural differences from the perspective of the GM network.

Based on the individual level, disruption of functional networks was found in ESRD patients, indicated by decreased global measures and abnormal nodal measures. Consistent with previous studies, we observed decreased γ and σ of the functional network in ESRD patients (Mu et al., 2018; Jin et al., 2021). A similar decline in separation capabilities in both GM and functional networks was found in our study. This finding may suggest a link between microstructural connectivity and functional connectivity in the brain of ESRD patients (Suárez et al., 2020). Moreover, decreased γ and σ of functional networks were positively correlated to MoCA scores in ESRD patients, which indicates that these parameters and potential imaging markers may reflect CI.

In contrast to our global measure findings, ESRD patients had more subtle changes in nodal measures of the functional network. Altered Ne and Dc were mainly observed in the default mode network, limbic system, frontal lobe, temporal lobe, and occipital lobe. This result was in accordance with previous studies and expanded our understanding of network organization degradation in ESRD patients (Ma S. et al., 2020; Wu et al., 2020; Yue et al., 2021). First, we observed a lateralized distribution of these brain regions. All brain regions with increased nodal parameters were included in the left cerebral hemisphere, and most brain regions with decreased nodal parameters were included in the right cerebral hemisphere. This may be related to the left-lateralization in righted-handed people (Morita et al., 2020). Our data strongly indicate that left regions of the brain may show more strong associations with brain compensatory mechanisms, and the right regions may be more susceptible to brain network impairment in right-handed ESRD patients. While our analyses could not directly address the relationship between alterations of nodal measures and pathophysiological changes, our results may be partly attributed to the ESRD-related compensatory mechanism. Second, decreased Ne of the functional network in the right insula and the right superior temporal gyrus showed a significantly positive correlation with MoCA scores. The insula is a part of the highly interconnected cognitive control network involved in various functional tasks, including auditory and vestibular functions, motor plasticity, cognitive control, and individual and social emotions (Nieuwenhuys, 2012). The superior temporal sulcus is a crucial hub for speech perception and emotional and memorial information processing (Nourski et al., 2021). Taken together, these data suggest that nodal properties of brain regions may be used as sensitive imaging markers to detect CI, and non-invasive neural intervention may be feasible in the clinic.

Although we observed significant correlations between network measures and MoCA scores in ESRD patients, there were limitations that limited the ability of these results to guide early clinical diagnoses based only on correlation analyses. Thus, we further applied optimized LSSVRM to tentatively predict cognitive function in ESRD patients. LSSVRM is an extension of the support vector regression machine (SVRM) that improves prediction efficiency by altering the inequality constraint and quadratic programming problems in the SVRM model into the equality constraint and solution of linear equations. Furthermore, the LSSVRM improves prediction accuracy by taking the error square and loss function to effectively fit the cognitive scores with non-linear characteristics (Liu et al., 2019). WOA can optimize the selection strategy of kernel function parameters and further improve the operating efficiency of the model (Zhang et al., 2018). The combination of LSSVRM and WOA takes work efficiency and prediction accuracy into account (Zhang et al., 2022). In our study, the prediction models based on functional networks showed a relatively great prediction accuracy, with the prediction accuracy based on combined global and nodal measures being slightly higher than that based only on a single type of measures. This may suggest that critical nodal changes have a certain influence on the overall CI observed in ESRD patients. Given that brain network changes were associated with cognitive performance and may be able to be selected as features to construct prediction models, it would be of further interest to investigate the feasibility of clinical application in future studies.

Despite the novelty of our study findings, we acknowledge some limitations of our study. First, due to the cross-sectional design and relatively small sample size, the power of the statistical analysis and prediction results may be affected. And the parameters of prediction models should be used with caution. Longitudinal studies with larger sample sizes are needed to provide dynamic perspectives in the future. Second, further neuropsychological tests are essential to provide more information. Third, exploring the occurrence and progression of CI will further contribute to the efficient development of clinical intervention. Thus, in our future researches, we will include ESRD patients with varying degrees of CI, including normal cognitive function, subjective cognitive function, mild CI, and moderate CI. Finally, the feature extraction, selection, and fusion methods utilized can be improved to better mine the information of structural and functional brain networks and enhance the prediction accuracy achieved.

## Conclusion

In summary, our study demonstrated that disrupted GM and functional networks in ESRD patients on maintenance hemodialysis contribute to and can be used to predict CI. In contrast to most diffusion MRI studies that focus merely on WM networks, our study paid attention to GM networks. Moreover, related functional network metrics were found to be significant imaging markers capable of predicting cognitive function in ESRD patients. Ultimately, this study highlighted the feasibility and necessity of early diagnosis of CI in ESRD patients from the perspective of quantitative imaging parameters.

## Data availability statement

The raw data supporting the conclusions of this article will be made available by the authors, without undue reservation.

## Ethics statement

The studies involving human participants were reviewed and approved by The Affiliated Changzhou No. 2 People’s Hospital of Nanjing Medical University. The patients/participants provided their written informed consent to participate in this study.

## Author contributions

JZ and HS made substantial contributions to the conception and design of this study. JZ and XW made substantial contributions to the acquisition of data. JZ made substantial contributions to the writing of manuscript. JD, TL, and ZJ made substantial contributions to the revision of the manuscript. All authors revised the draft for intellectual content, gave their final approval of the final version for publication, and agreed to be accountable for all aspects of the work in ensuring that questions related to the accuracy or integrity of any part of this study were appropriately investigated and resolved.

## Footnotes

1
https://fsl.fmrib.ox.ac.uk/fsl

2
https://www.nitrc.org/projects/dke/

3
https://www.fil.ion.ucl.ac.uk/spm/

4
http://rfmri.org/dpabi

5
http://www.nitrc.org/projects/gretna/

## References

[B1] Agganis B. T. Weiner D. E. Giang L. M. Scott T. Tighiouart H. Griffith J. L. (2010). Depression and cognitive function in maintenance hemodialysis patients. *Am. J. Kidney Dis.* 56 704–712. 10.1053/j.ajkd.2010.04.018 20673602 PMC2943330

[B2] Barkhof F. Haller S. Rombouts S. A. (2014). Resting-state functional MR imaging: A new window to the brain. *Radiology* 272 29–49. 10.1148/radiol.14132388 24956047

[B3] Basser P. J. Mattiello J. LeBihan D. (1994). MR diffusion tensor spectroscopy and imaging. *Biophys. J.* 66 259–267. 10.1016/S0006-3495(94)80775-1 8130344 PMC1275686

[B4] Bingbing G. Yujing Z. Yanwei M. Chunbo D. Weiwei W. Shiyun T. (2020). Diffusion kurtosis imaging of microstructural changes in gray matter nucleus in Parkinson disease. *Front. Neurol.* 11:252. 10.3389/fneur.2020.00252 32362865 PMC7180218

[B5] Bugnicourt J. Godefroy O. Chillon J. Choukroun G. Massy Z. A. (2013). Cognitive disorders and dementia in CKD: The neglected kidney-brain axis. *J. Am. Soc. Nephrol.* 24 353–363. 10.1681/ASN.2012050536 23291474

[B6] Bullmore E. Sporns O. (2009). Complex brain networks: Graph theoretical analysis of structural and functional systems. *Nat. Rev. Neurosci.* 10 186–198. 10.1038/nrn2575 19190637

[B7] Bullmore E. Sporns O. (2012). The economy of brain network organization. *Nat. Rev. Neurosci.* 13 336–349. 10.1038/nrn3214 22498897

[B8] Chandra A. Dervenoulas G. Politis M. (2019). Magnetic resonance imaging in Alzheimer’s disease and mild cognitive impairment. *J. Neurol.* 266 1293–1302. 10.1007/s00415-018-9016-3 30120563 PMC6517561

[B9] Chen H. J. Qiu J. Fu Q. Chen F. (2020). Alterations of spontaneous brain activity in hemodialysis patients. *Front. Hum. Neurosci.* 14:278. 10.3389/fnhum.2020.00278 32765243 PMC7381103

[B10] Cheng J. Zhang H. Peng Z. Xu Y. Tang H. Wu J. (2018). Divergent topological networks in Alzheimer’s disease: A diffusion kurtosis imaging analysis. *Transl. Neurodegener.* 7:10. 10.1186/s40035-018-0115-y 29719719 PMC5921324

[B11] Chou M. C. Ko C. H. Chang J. M. Hsieh T. J. (2019). Disruptions of brain structural network in end-stage renal disease patients with long-term hemodialysis and normal-appearing brain tissues. *J. Neuroradiol.* 46 256–262. 10.1016/j.neurad.2018.04.004 29733919

[B12] Donzuso G. Monastero R. Cicero C. E. Luca A. Mostile G. Giuliano L. (2021). Neuroanatomical changes in early Parkinson’s disease with mild cognitive impairment: A VBM study; the Parkinson’s disease cognitive impairment study (PaCoS). *Neurol. Sci.* 42 3723–3731. 10.1007/s10072-020-05034-9 33447925

[B13] Faskhodi M. M. Einalou Z. Dadgostar M. (2018). Diagnosis of Alzheimer’s disease using resting-state fMRI and graph theory. *Technol. Health Care* 26 921–931. 10.3233/THC-181312 30124458

[B14] Friston K. (2002). Beyond phrenology: What can neuroimaging tell us about distributed circuitry? *Annu. Rev. Neurosci.* 25 221–250. 10.1146/annurev.neuro.25.112701.142846 12052909

[B15] Goghari V. M. Kusi M. Shakeel M. K. Beasley C. David S. Leemans A. (2021). Diffusion kurtosis imaging of white matter in bipolar disorder. *Psychiatry Res. Neuroimaging* 317:111341. 10.1016/j.pscychresns.2021.111341 34411810

[B16] Guo H. Liu W. Li H. Yang J. (2021). Structural and functional brain changes in hemodialysis patients with end-stage renal disease: DTI analysis results and ALFF analysis results. *Volume* 14 77–86. 10.2147/IJNRD.S295025 33727853 PMC7955761

[B17] Hempel J. Brendle C. Adib S. D. Behling F. Tabatabai G. Castaneda Vega S. (2021). Glioma-specific diffusion signature in diffusion kurtosis imaging. *J. Clin. Med.* 10:2325. 10.3390/jcm10112325 34073442 PMC8199055

[B18] Hu R. Gao L. Chen P. Wu B. Wu X. Xu H. (2020). How do you feel now? The salience network functional connectivity in end-stage renal disease. *Front Neurosci.* 14:533910. 10.3389/fnins.2020.533910 33304233 PMC7693456

[B19] Hui E. S. Cheung M. M. Qi L. Wu E. X. (2008). Advanced MR diffusion characterization of neural tissue using directional diffusion kurtosis analysis. *Annu. Int. Conf. IEEE Eng. Med. Biol. Soc.* 2008 3941–3944. 10.1109/IEMBS.2008.4650072 19163575

[B20] Jensen J. H. Helpern J. A. Ramani A. Lu H. Kaczynski K. (2005). Diffusional kurtosis imaging: The quantification of non-gaussian water diffusion by means of magnetic resonance imaging. *Magn. Reson. Med.* 53 1432–1440. 10.1002/mrm.20508 15906300

[B21] Jiang W. Li J. Chen X. Ye W. Zheng J. (2017). Disrupted structural and functional networks and their correlation with alertness in right temporal lobe epilepsy: A graph theory study. *Front. Neurol.* 8:179. 10.3389/fneur.2017.00179 28515708 PMC5413548

[B22] Jin M. Wang L. Wang H. Han X. Diao Z. Guo W. (2020). Structural and functional alterations in hemodialysis patients: A voxel-based morphometry and functional connectivity study. *Front. Hum. Neurosci.* 14:80. 10.3389/fnhum.2020.00080 32218727 PMC7078368

[B23] Jin M. Wang L. Wang H. Han X. Diao Z. Guo W. (2021). Altered resting-state functional networks in patients with hemodialysis: A graph-theoretical based study. *Brain Imaging Behav.* 15 833–845. 10.1007/s11682-020-00293-8 32314197

[B24] Kalantar-Zadeh K. Jafar T. H. Nitsch D. Neuen B. L. Perkovic V. (2021). Chronic kidney disease. *Lancet* 398 786–802. 10.1016/S0140-6736(21)00519-5 34175022

[B25] Li P. Ding D. Ma X. Zhang H. Liu J. Zhang M. (2018). Altered intrinsic brain activity and memory performance improvement in patients with end-stage renal disease during a single dialysis session. *Brain Imaging Behav.* 12 1640–1649. 10.1007/s11682-018-9828-x 29374356

[B26] Li P. Mu J. Ma X. Ding D. Ma S. Zhang H. (2021). Neurovascular coupling dysfunction in end-stage renal disease patients related to cognitive impairment. *J. Cereb. Blood Flow Metab.* 41 2593–2606. 10.1177/0271678X211007960 33853410 PMC8504946

[B27] Liu J. Zheng H. Zhang Y. Li X. Fang J. Liu Y. (2019). Dissolved gases forecasting based on wavelet least squares support vector regression and imperialist competition algorithm for assessing incipient faults of transformer polymer insulation. *Polymers* 11:85. 10.3390/polym11010085 30960069 PMC6402009

[B28] Luo W. Jiang X. Wei X. Li S. Li M. (2015). Retracted article: A study on cognitive impairment and gray matter volume abnormalities in silent cerebral infarction patients. *Neuroradiology* 57 783–789. 10.1007/s00234-015-1535-3 25903433

[B29] Ma C. Tian F. Ma M. Su H. Fan J. Li Z. (2020). Preferentially disrupted core hubs within the default-mode network in patients with end-stage renal disease: A resting-state functional magnetic resonance imaging study. *Front. Neurol.* 11:1032. 10.3389/fneur.2020.01032 33250836 PMC7674924

[B30] Ma S. Zhang M. Liu Y. Ding D. Li P. Ma X. (2020). Abnormal rich club organization in end-stage renal disease patients before dialysis initiation and undergoing maintenance hemodialysis. *BMC Nephrol.* 21:515. 10.1186/s12882-020-02176-y 33243163 PMC7689979

[B31] McIntosh A. R. (2000). Towards a network theory of cognition. *Neural Netw.* 13 861–870. 10.1016/S0893-6080(00)00059-9 11156197

[B32] McKenna F. F. Miles L. Babb J. S. Goff D. C. Lazar M. (2019). Diffusion kurtosis imaging of gray matter in schizophrenia. *Cortex* 121 201–224. 10.1016/j.cortex.2019.08.013 31629198 PMC7556324

[B33] Morita T. Asada M. Naito E. (2020). Right-hemispheric dominance in self-body recognition is altered in left-handed individuals. *Neuroscience* 425 68–89. 10.1016/j.neuroscience.2019.10.056 31809726

[B34] Mu J. Chen T. Liu Q. Ding D. Ma X. Li P. (2018). Abnormal interaction between cognitive control network and affective network in patients with end-stage renal disease. *Brain Imaging Behav.* 12 1099–1111. 10.1007/s11682-017-9782-z 29063504

[B35] Mu J. Ma L. Ding D. Ma X. Li P. Li R. (2021). White matter characteristics between amygdala and prefrontal cortex underlie depressive tendency in end stage renal disease patients before the dialysis initiation. *Brain Imaging Behav.* 15 1815–1827. 10.1007/s11682-020-00376-6 33048290

[B36] Nasreddine Z. S. Phillips N. A. Bedirian V. Charbonneau S. Whitehead V. Collin I. (2005). The montreal cognitive assessment. MoCA: A brief screening tool for mild cognitive impairment. *J. Am. Geriatr. Soc.* 53 695–699. 10.1111/j.1532-5415.2005.53221.x 15817019

[B37] Natale P. Palmer S. C. Ruospo M. Saglimbene V. M. Rabindranath K. S. Strippoli G. F. (2019). Psychosocial interventions for preventing and treating depression in dialysis patients. *Cochrane Database Syst. Rev.* 12:D4542. 10.1002/14651858.CD004542.pub3 31789430 PMC6886341

[B38] Nieuwenhuys R. (2012). The insular cortex: A review. *Prog. Brain Res.* 195 123–163. 10.1016/B978-0-444-53860-4.00007-6 22230626

[B39] Nourski K. V. Steinschneider M. Rhone A. E. Kovach C. K. Banks M. I. Krause B. M. (2021). Electrophysiology of the human superior temporal sulcus during speech processing. *Cereb. Cortex* 31 1131–1148. 10.1093/cercor/bhaa281 33063098 PMC7786351

[B40] Park B. S. Kim S. E. Lee H. Kim Y. W. Kim I. H. Park J. H. (2020). Alterations in structural and functional connectivities in patients with end-stage renal disease. *J. Clin. Neurol.* 16:390. 10.3988/jcn.2020.16.3.390 32657059 PMC7354985

[B41] Pezzoli S. Sánchez-Valle R. Solanes A. Kempton M. J. Bandmann O. Shin J. I. (2021). Neuroanatomical and cognitive correlates of visual hallucinations in Parkinson’s disease and dementia with Lewy bodies: Voxel-based morphometry and neuropsychological meta-analysis. *Neurosci. Biobehav. Rev.* 128 367–382. 10.1016/j.neubiorev.2021.06.030 34171324

[B42] Reijneveld J. C. Ponten S. C. Berendse H. W. Stam C. J. (2007). The application of graph theoretical analysis to complex networks in the brain. *Clin. Neurophysiol.* 118 2317–2331. 10.1016/j.clinph.2007.08.010 17900977

[B43] Rubinov M. Sporns O. (2010). Complex network measures of brain connectivity: Uses and interpretations. *Neuroimage* 52 1059–1069. 10.1016/j.neuroimage.2009.10.003 19819337

[B44] Shi M. Liu S. Chen H. Geng W. Yin X. Chen Y. (2021). Disrupted brain functional network topology in unilateral acute brainstem ischemic stroke. *Brain Imaging Behav.* 15 444–452. 10.1007/s11682-020-00353-z 32705464

[B45] Stenberg J. Eikenes L. Moen K. G. Vik A. Håberg A. K. Skandsen T. (2021). Acute diffusion tensor and kurtosis imaging and outcome following mild traumatic brain injury. *J. Neurotrauma* 38 2560–2571. 10.1089/neu.2021.0074 33858218 PMC8403189

[B46] Steven A. J. Zhuo J. Melhem E. R. (2014). Diffusion kurtosis imaging: An emerging technique for evaluating the microstructural environment of the brain. *Am. J. Roentgenol.* 202:W26. 10.2214/AJR.13.11365 24370162

[B47] Suárez L. E. Markello R. D. Betzel R. F. Misic B. (2020). Linking structure and function in macroscale brain networks. *Trends Cogn. Sci.* 24 302–315. 10.1016/j.tics.2020.01.008 32160567

[B48] Tabesh A. Jensen J. H. Ardekani B. A. Helpern J. A. (2011). Estimation of tensors and tensor-derived measures in diffusional kurtosis imaging. *Magn. Reson. Med.* 65 823–836. 10.1002/mrm.22655 21337412 PMC3042509

[B49] van Zwieten A. Wong G. Ruospo M. Palmer S. C. Barulli M. R. Iurillo A. (2018). Prevalence and patterns of cognitive impairment in adult hemodialysis patients: The COGNITIVE-HD study. *Nephrol. Dial. Transpl.* 33 1197–1206. 10.1093/ndt/gfx314 29186522

[B50] Wu B. Li X. Zhang M. Zhang F. Long X. Gong Q. (2020). Disrupted brain functional networks in patients with end-stage renal disease undergoing hemodialysis. *J. Neurosci. Res.* 98 2566–2578. 10.1002/jnr.24725 32930417

[B51] Yue Z. Wang P. Li X. Ren J. Wu B. (2021). Abnormal brain functional networks in end-stage renal disease patients with cognitive impairment. *Brain Behav.* 11:e02076. 10.1002/brb3.2076 33605530 PMC8035483

[B52] Zhang J. Cortese R. De Stefano N. Giorgio A. (2021). Structural and functional connectivity substrates of cognitive impairment in multiple sclerosis. *Front. Neurol.* 12:671894. 10.3389/fneur.2021.671894 34305785 PMC8297166

[B53] Zhang L. Wu H. Zhang A. Bai T. Ji G. Tian Y. (2021). Aberrant brain network topology in the frontoparietal-limbic circuit in bipolar disorder: A graph-theory study. *Eur. Arch. Psy. Clin. Neurosci.* 271 1379–1391. 10.1007/s00406-020-01219-7 33386961

[B54] Zhang Y. Liu X. Hou Z. Yin Y. Xie C. Zhang H. (2021). Global topology alteration of the brain functional network affects the 8-week antidepressant response in major depressive disorder. *J. Affect. Disord.* 294 491–496. 10.1016/j.jad.2021.07.078 34330044

[B55] Zhang Y. Wang S. Sui Y. Yang M. Liu B. Cheng H. (2018). Multivariate approach for Alzheimer’s disease detection using stationary wavelet entropy and predator-prey particle swarm optimization. *J. Alzheimer’s Dis.* 65 855–869. 10.3233/JAD-170069 28731432

[B56] Zhang Y. Xi Z. Zheng J. Shi H. Jiao Z. (2022). GWLS: A novel model for predicting cognitive function scores in patients with end-stage renal disease. *Front. Aging Neurosci.* 14:834331. 10.3389/fnagi.2022.834331 35185530 PMC8850953

[B57] Zheng G. Wen J. Yu W. Li X. Zhang Z. Chen H. (2016). Anemia rather than hypertension contributes to cerebral hyperperfusion in young adults undergoing hemodialysis: A phase contrast MRI study. *Sci Rep.* 6:22346. 10.1038/srep22346 26923866 PMC4770317

